# Severe *Chlamydia psittaci* pneumonia complicated by deep vein thrombosis: a case report

**DOI:** 10.3389/fmed.2025.1527556

**Published:** 2025-02-25

**Authors:** Anbing Zhang, Ting Huang, Xiaoli Lao, Jun Ma, Xiuqiong Xia, Jianping Liang

**Affiliations:** ^1^Department of Pulmonary and Critical Care Medicine, Zhongshan People’s Hospital, Zhongshan, China; ^2^Shenzhen University Medical School, Shenzhen University, Shenzhen, China; ^3^Graduate School, Guangdong Medical University, Zhanjiang, China

**Keywords:** psittacosis, next-generation sequencing, D-dimer, anticoagulant, deep vein thrombosis

## Abstract

Deep vein thrombosis (DVT) of the legs is a rare but clinically important complication of *Chlamydia psittaci* pneumonia. We report a case of a 51-year-old man who was admitted to the hospital with fever, cough, and dyspnea. Next-generation sequencing confirmed the diagnosis of *Chlamydia psittaci* pneumonia. His D-dimer level was elevated on admission, and ultrasound confirmed DVT in the legs. The patient was treated with intravenous doxycycline for the infection and rivaroxaban as an anticoagulant. His condition gradually improved and he was discharged after making a full recovery. In this paper, we explore the potential association between *Chlamydia psittaci* infection and venous thrombosis, as well as clinical management strategies.

## Introduction

*Chlamydia psittaci* is a pathogen commonly found in birds that can also infect humans, causing psittacosis (1). In humans, psittacosis typically manifests as pneumonia, with clinical symptoms including fever, cough, and dyspnea. In severe cases, it can lead to life-threatening pneumonia (2). Although psittacosis is uncommon, the risk of transmission is increasing owing to globalization and the growing trade of poultry and pet birds (2).

Surgery, fractures, and cancer are common causes of venous thromboembolism (VTE), whereas infection-induced VTE is relatively rare (3). Deep vein thrombosis (DVT) of the lower legs and pulmonary thromboembolism are seldom reported in cases of *Chlamydia psittaci* infection. Fang and Xu (4) reported a case of pulmonary thrombosis induced by psittacosis pneumonia, suggesting a possible association between psittacosis and pulmonary thrombosis; however, this hypothesis requires further clinical evidence. He et al. (5) described a case of *Chlamydia psittaci* pneumonia complicated by atherosclerosis obliterans of the legs. Pulmonary thrombosis and venous thrombosis are pathophysiological processes linked to stasis, vascular endothelial injury, and hypercoagulability. The risk of these conditions is increased in individuals who are bedridden, recovering from surgery, and those with cancer (6). However, severe infections can lead to an excessive inflammatory response, causing endothelial injury and hypercoagulable states, thereby increasing the risk of pulmonary thrombosis and venous thrombosis (7).

We describe a rare case of *Chlamydia psittaci* pneumonia complicated by DVT of the legs. We performed a detailed case analysis to explore the potential association between *Chlamydia psittaci* infection and venous thrombosis and provide a source of reference for clinical diagnosis and treatment.

## Case description

The patient was a 51-year-old man who was admitted to hospital with a 5-day history of fever, cough, and dyspnea. He had no known underlying medical conditions. Five days previously, he had developed a persistent high fever (with a maximum temperature of 40°C), accompanied by cough and shortness of breath. On admission, physical examination revealed a temperature of 39°C, blood pressure of 138/98 mmHg, heart rate of 116 bpm, respiratory rate of 29 breaths/min, and oxygen saturation of 89% breathing room air. Auscultation of the lungs revealed coarse breath sounds and scattered moist rales. No peripheral edema was noted in his lower legs.

**FIGURE 1:**
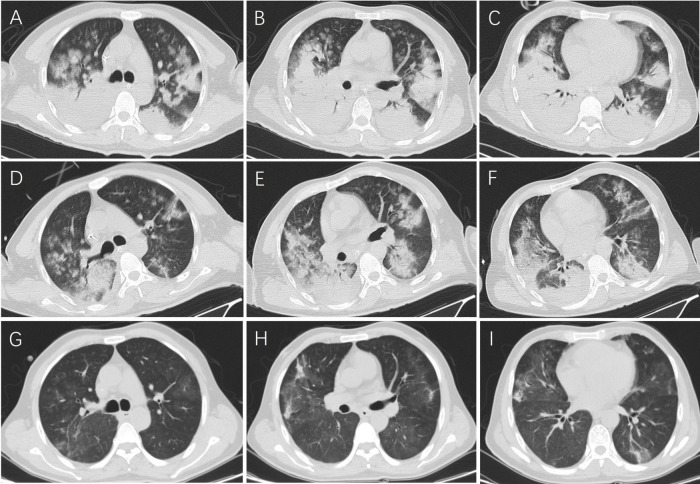
Chest computed tomography (CT) of the patient: The initial chest CT on admission showed bilateral pneumonia **(A–C)**. After 1 week of doxycycline treatment, the chest CT showed partial absorption of the pneumonia **(D–F)**. After 3 weeks of doxycycline treatment, the chest CT showed marked absorption of the pneumonia **(G–I)**.

Laboratory tests on admission showed an elevated white blood cell, neutrophil, and platelet counts, and elevated D-dimer, C-reactive protein (CRP), procalcitonin, and interleukin-6 (IL-6) levels (Table 1). Arterial blood gas analysis showed pH 7.441, PO*2* 59.1 mmHg, PCO*2* 26.5 mmHg, and HCO*3* 20.6 mmol/L. Liver function tests revealed elevated alanine aminotransferase (ALT) and aspartate aminotransferase (AST) levels, Blood biochemistry revealed low total protein (57.1 g/L) and albumin (31.6 g/L) levels. His electrolyte levels were as follows: potassium 4.26 mmol/L, sodium 148 mmol/L, chloride 113 mmol/L, calcium 2.14 mmol/L, and phosphorus 0.8 mmol/L. Renal function tests showed a creatinine level of 68 μmol/L, uric acid level of 71 μmol/L, and urea level of 8.31 mmol/L. His creatine kinase (CK) level was 32 U/L, creatine kinase MB isoenzyme (CK-MB) level was 8 U/L, N-terminal-pro-brain natriuretic peptide (NT-proBNP) level was 60 pg/mL, and fibrinogen level was 3.2 g/L. Nucleic acid tests for influenza and SARS-CoV-2, a dengue antigen test, and a *Mycoplasma pneumoniae* antibody test all were negative. Chest computed tomography (CT) revealed extensive consolidation in both lungs and a small amount of pleural effusion on the right side (Figures 1A–C).

**TABLE 1:** The patient’s laboratory test results.

Test parameter	Normal range	On the day of admission	2 weeks after starting DOX
WBC count (×10^9^/L)	4–10	8.8	7.76
Neutrophil count (×10^9^/L)	2–7	7.3	5.47
Neutrophil (%)	40–75	83	74
Lymphocyte count (×10^9^/L)	0.8–4	0.76	1.21
Lymphocyte (%)	20–40	8.6	15.6
Hemoglobin (g/L)	113–151	140	143
Platelets (×10^9^/L)	101–320	407	240
CRP (mg/L)	0–8	97	5.14
PCT (ng/mL)	0–5	0.57	0.06
D-dimer (mg/L)	0–0.5	6.53	1.61
IL-6 (pg/mL)	0-7	197	9.73
ALT (U/L)	7–40	45	26
AST (U/L)	13–40	49	33
Creatinine (μmol/L)	45–84	68	50
CK (U/L)	26–174	32	28
K (mmol/L)	3.5–5.5	4.26	3.9
Na (mmol/L)	135–145	148	134
Cl (mmol/L)	95–105	113	98
Fibrinogen (g/L)	2–4	3.2	2.59
NGS *C. psittaci* reads (n)	/	85915	/

ALT, alanine aminotransferase; AST, aspartate aminotransferase; DOX, doxycycline; CK, creatine kinase; CRP, C-reactive protein; IL-6, interleukin-6; NGS, next-generation sequencing, PCT, procalcitonin; WBC, white blood cells.

The patient was immediately intubated in the emergency room and mechanical ventilation was initiated. He was admitted to the intensive care unit and started on intravenous cefoperazone-sulbactam (1.5 g, 8-hourly) for infection control. Owing to the severity of his condition and the possibility of infection by a rare pathogen, bronchoscopy and bronchoalveolar lavage of the right lower lobe were performed on the day of admission. A sample of bronchoalveolar lavage fluid (BALF) was sent for next-generation sequencing (NGS). The next day, NGS identified *Chlamydia psittaci* (with 85,915 sequence reads) in the BALF. Further questioning revealed that the patient had a history of slaughtering poultry at home and had slaughtered a chicken 1 week prior to the onset of his symptoms. He was diagnosed with severe *Chlamydia psittaci* pneumonia and the antibiotic was switched to intravenous doxycycline (0.1 g, 12-hourly). Three days after starting doxycycline treatment, his fever resolved.

**FIGURE 2:**
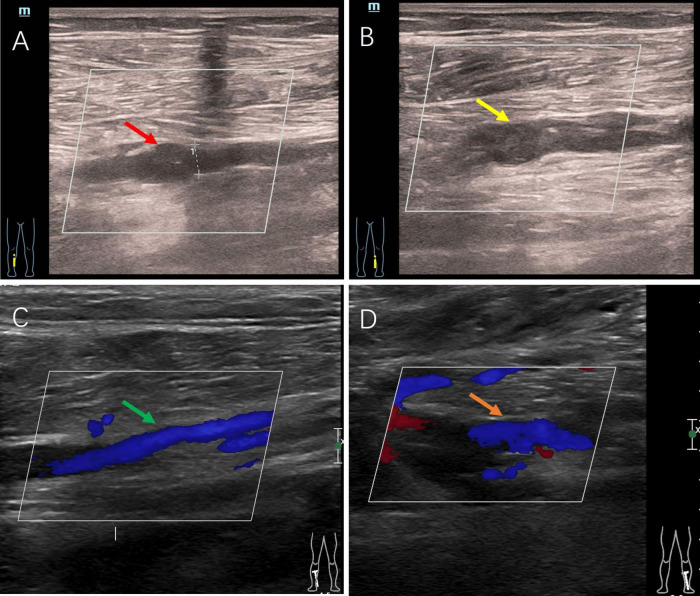
Venous ultrasound of the legs on admission showing intermuscular venous thrombosis in the right calf (**(A)**, red arrows); and left calf **(B)**, yellow arrows). Follow-up venous ultrasound of the legs after 2 months of rivaroxaban therapy revealed complete resolution of the intermuscular vein thrombosis (right calf, **(C)**, green arrows; and left calf, **(D)**, orange arrows).

On admission, the patient’s D-dimer level was considerably elevated (6.53 mg/L; reference < 0.5 mg/L), which made us consider the possibility of a clotting condition. Although he had no obvious swelling in his lower legs, a venous ultrasound was performed of both legs. This revealed thrombosis and complete occlusion in the intermuscular veins of both legs (Figures 2A, B). However, the patient’s family refused pulmonary artery computed tomography angiography (CTA), so the presence of pulmonary thromboembolism could not be investigated. The patient was diagnosed with DVT of the legs and oral rivaroxaban (15 mg, twice daily) was initiated. The dose was reduced to 20 mg daily after 3 weeks.

Follow-up chest CT 1 week after starting doxycycline treatment revealed reduced lung consolidation and pleural effusion (Figures 1D–F). The patient was extubated and transferred to a general ward. Doxycycline therapy was continued and his symptoms improved after 1 week. He recovered and was discharged. Oral doxycycline (0.1 g/dose, orally, twice daily for 2 weeks) and rivaroxaban treatment were continued following discharge. Follow-up chest CT after 3 weeks of doxycycline therapy revealed marked improvement of the lung lesions (Figures 1G–I), and his D-dimer levels decreased to within the normal range. Follow-up venous ultrasound of the legs after 2 months of rivaroxaban therapy revealed complete resolution of the intermuscular vein thrombosis (Figures 2C, D).

## Discussion

This report describes a rare case of *Chlamydia psittaci* pneumonia complicated by DVT of the legs. Psittacosis is a zoonosis and is primarily transmitted through contact with respiratory secretions, feces, and other excreta from poultry and other birds (8). However, our patient did not have an obvious history of bird contact, such as poultry rearing, and it was only on detailed questioning after receiving the NGS results showing *Chlamydia psittaci* in the BALF that the history of poultry exposure was discovered. This highlights the importance of detailed history-taking so as not to overlook atypical exposures. The clinical manifestations of *Chlamydia psittaci* infection are varied and can easily be confused with other types of pneumonia. Early and accurate identification of the infecting pathogen is critical for timely and effective treatment. In patients with community-acquired pneumonia that does not respond to empirical antibiotic treatment, NGS of BALF should be performed to identify the pathogen. The antibiotic treatment should be adjusted accordingly once the pathogen has been identified (9).

The main clinical manifestation of *Chlamydia psittaci* infection is pneumonia (2). Owing to the nonspecific clinical manifestations, it can easily be mistaken for pneumonia caused by other pathogens, which may lead to misdiagnosis or missed diagnosis. In this case, the patient initially presented with high fever, cough, and dyspnea, symptoms similar to common bacterial infections. However, he did not respond to standard broad-spectrum antibiotic treatment, leading us to suspect infection by an atypical pathogen. Ultimately, NGS confirmed *Chlamydia psittaci* infection. This highlights the importance of considering rare pathogens such as *Chlamydia psittaci* in cases of unexplained pneumonia.

In this case, the patient developed DVT of the legs during the course of *Chlamydia psittaci* infection. Although an association has been reported between infectious diseases and venous thrombosis (10–12), cases of psittacosis complicated by pulmonary thrombosis or DVT are rare (4). The high incidence of thromboembolic events in patients hospitalized with COVID-19 has provided evidence of the effectiveness of anticoagulant therapy in infection-induced thrombosis (13, 14). COVID-19 is closely associated with venous thromboembolic diseases, including deep vein thrombosis and pulmonary embolism (15). Previous studies have shown that SARS-CoV-2 infection reduces the expression of ACE2 molecules on the cell surface, which may lead to activation of the renin-angiotensin system, promoting vascular constriction and endothelial injury. Endothelial injury, in turn, results in upregulated expression of tissue factors and an imbalance in the fibrinolytic system (16). *Chlamydia pneumoniae* infection may increase the risk of thrombosis by activating platelets (17), and may contribute to the development of atherosclerosis by inducing chronic inflammation (18). The exact mechanisms by which infection triggers thrombosis are not fully understood but may involve activation of inflammation, endothelial cell injury, autoimmune responses, and an imbalance between coagulation and anticoagulation (19, 20). In addition, infection can potentially cause VTE as a result of reduced mobility. Epaulard (21) reviewed previous research which showed that the risk of VTE during infection is mediated by the link between inflammation and activation of coagulation. IL-6 and CRP are nonspecific markers of inflammation and tissue injury (15). Studies have shown that elevated levels of IL-6 and CRP can promote pulmonary thrombosis (22). High CRP levels are also associated with an increased risk of VTE (23). CRP influences tissue factor synthesis, hemostasis activation, and fibrinolysis (24, 25), reflecting underlying inflammation or hypercoagulable states that may contribute to the occurrence of VTE (26). Although elevated IL-6 and CRP levels are seen in other conditions, their combination with an elevated D-dimer level led us to suspect VTE in this case. Fibrinogen can increase blood viscosity, thus increasing the risk of thrombosis (19).

Early recognition of VTE can be difficult when patients do not exhibit classic symptoms such as hemoptysis, chest pain, or swelling of the calves; therefore, monitoring of D-dimer levels is particularly important. Our patient had elevated D-dimer levels on admission, prompting immediate venous ultrasound examination of his legs, which confirmed the diagnosis of DVT. The patient’s family declined pulmonary artery CTA, so it was not possible to determine whether the patient had an associated pulmonary embolism. D-dimer testing is essential for patients clinically at high risk of VTE, as a negative D-dimer result can rule out acute pulmonary embolism (27).

In managing this case, the patient was immediately started on anticoagulant therapy after the DVT diagnosis, and doxycycline was used for infection control. This combined treatment strategy controlled the infection and prevented further expansion of the thrombosis and related complications. Anticoagulant therapy is crucial for patients with DVT; however, the benefit of anticoagulants must be carefully weighed against the risks. Novel anticoagulants such as rivaroxaban, dabigatran, argatroban, bivalirudin, apixaban, and edoxaban offer stronger anticoagulant effects with a lower risk of bleeding compared with traditional anticoagulants, and have recently become the preferred option for antithrombotic therapy (28). Compared with traditional anticoagulants such as warfarin, rivaroxaban is easier to administer, has more rapid onset of effect, and does not require monitoring (19). Rivaroxaban reduces the size of the thrombus and the risk of recurrence, without increasing the risk bleeding, making it a preferred option over standard anticoagulants (29).

## Conclusion

*Chlamydia psittaci* pneumonia complicated by venous thromboembolism is extremely rare. In the absence of symptoms and signs such as chest pain, hemoptysis, and swelling of the lower legs, D-dimer monitoring can aid in the early identification of venous thromboembolism. Timely detection of thrombosis and initiation of anticoagulant therapy can improve the outcome.

## Data Availability

The datasets presented in this article are not readily available because of ethical/privacy restrictions. Requests to access the datasets should be directed to the corresponding authors.
